# Roles, Regulation, and Agricultural Application of Plant Phosphate Transporters

**DOI:** 10.3389/fpls.2017.00817

**Published:** 2017-05-18

**Authors:** Duoliya Wang, Sulian Lv, Ping Jiang, Yinxin Li

**Affiliations:** ^1^Key Laboratory of Plant Molecular Physiology, Institute of Botany, Chinese Academy of SciencesBeijing, China; ^2^University of Chinese Academy of SciencesBeijing, China

**Keywords:** crop, *Eutrema salsugineum*, drought, genetic modification, low phosphate, phosphate transporter, regulation, salinity

## Abstract

Phosphorus (P) is an essential mineral nutrient for plant growth and development. Low availability of inorganic phosphate (orthophosphate; Pi) in soil seriously restricts the crop production, while excessive fertilization has caused environmental pollution. Pi acquisition and homeostasis depend on transport processes controlled Pi transporters, which are grouped into five families so far: PHT1, PHT2, PHT3, PHT4, and PHT5. This review summarizes the current understanding on plant PHT families, including phylogenetic analysis, function, and regulation. The potential application of Pi transporters and the related regulatory factors for developing genetically modified crops with high phosphorus use efficiency (PUE) are also discussed in this review. At last, we provide some potential strategies for developing high PUE crops under salt or drought stress conditions, which can be valuable for improving crop yields challenged by global scarcity of water resources and increasing soil salinization.

## Introduction

Phosphorus (P), one of the major plant macronutrients, is a structural component of nucleic acids and phospholipids and plays essential roles in energy transfer, signal transduction, and enzyme activation. P exists in two kinds of chemical forms in soil: organic form and inorganic form (orthophosphate, Pi). As the only form that can be assimilated by plant, Pi is commonly present at low concentration (less than 2 μM) in soil solution, even in fertile soil (10 μM), due to its uneven distribution, relative immobile and high fixation (Mimura, 1999; Raghothama, 1999). Low Pi availability has been a worldwide constraint for crop growth. Nowadays, phosphate fertilizer application is a conventional method to improve the Pi availability. However, only 10–20% of applied Pi can be absorbed by plants and overuse of phosphate fertilizer not only has been impeded by the non-renewable resource, phosphate rock, but also causes the water eutrophication (Holford, 1997; Oelkers and Valsami-Jones, 2008). Therefore, it is urgent and imperative to understand the plant Pi acquisition and distribution mechanism and enhance the crop phosphorus use efficiency (PUE). Plants have developed elaborate mechanisms to enhance Pi availability and assimilation. One adaptation to P deficiency commonly observed in most species is an increase in root surface area by formation of finer roots, aerenchyma, and root hairs, which would improve soil exploration (Lynch, 2007; Nestler et al., 2016). In addition, some plant species can secrete organic acids to release Pi through complexation reaction of organic acids with Al^3+^, Fe^3+^, Ca^2+,^ or phosphatases to release Pi from the organic sources, thus to enhance the availability of Pi in soils (López-Arredondo et al., 2014). Plant uptake and translocation of available Pi occurs through the concerted action of various Pi transporters. Based on the sequence identity and their varied subcellular localization, plant Pi transporters are grouped into five phylogenetically distinct classes of families: PHT1, PHT2, PHT3, PHT4, and PHT5 (Mudge et al., 2002; Rausch and Bucher, 2002; Liu T.Y. et al., 2016; Mlodzińska and Zboińska, 2016). In this review, we summarize the current state of research on plant PHT families, including phylogenetic analysis, function, and regulation. The agricultural application aspects of Pi transporters and regulatory factors are also discussed here. We also provide some potential strategies for improving crop PUE under salt or drought stress conditions.

## Phylogenetic Analysis and Roles of Plant Pi Transporters

**FIGURE 1:**
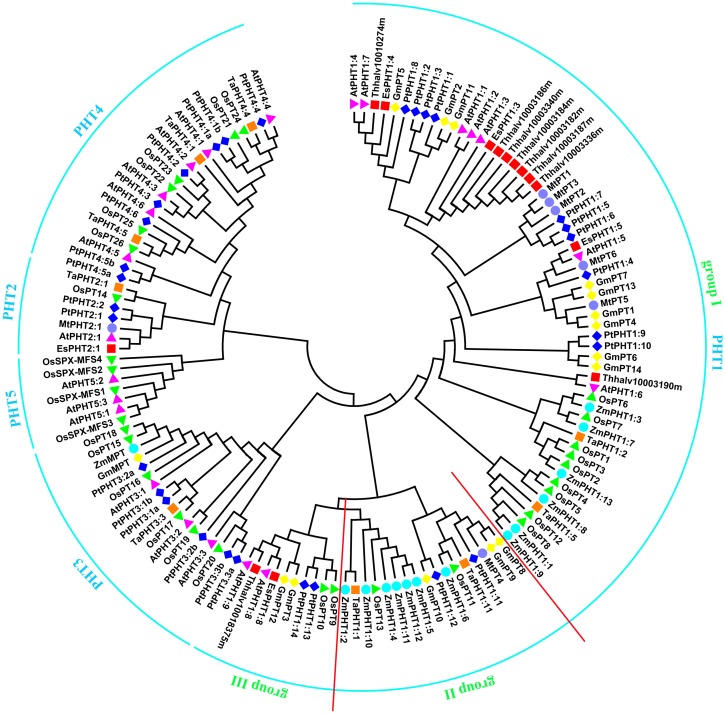
**Phylogenetic analysis of Pi transporters.** The phylogenetic tree was constructed by the Neighbor-Joining method with MEGA6.0, bootstrap values are from 1000 replicates. Accession numbers or locus IDs of all used proteins were given in Supplementary Table S1.

Utilizing the identified Pi transporter amino acid sequences from *Arabidopsis*, rice, soybean, maize, wheat, poplar, *Medicago truncatula* as well as a halophyte *E. salsugineum*, we constructed a phylogenetic tree using the neighbor-joining method with MEGA 6 program. Phylogenetic analysis showed that all the divergence of five phosphate transporter families preceded divergence of monocots and dicots and the members of PHT1, PHT2, PHT3, PHT4, and PHT5 clustered separately (**Figure 1**). The putative localization, tissue expression and conserved sequence of Pi transporters in *Arabidopsis* were summarized in **Table 1**. In general, plasma membrane (PM) located PHT1s mainly function in Pi acquisition from soil, whereas Pi distribution within the plant such as translocation against chloroplasts, mitochondria, Golgi, and vacuole is mediated by PHT2, PHT3, PHT4, and PHT5 family members. The functions of these PHT families in *Arabidopsis* and rice were summarized in a recent review by Mlodzińska and Zboińska (2016). In this section, we draw more attention to the identification and function of Pi transporters in other plants, especially in some important crops, attempting to throw light on the underlying Pi uptake and distribution mechanisms in these crops and to find potential genetic target for crop improvement breeding.

**Table 1:** Putative localization, tissue expression, and conserved sequence of Pi transporters in *Arabidopsis*.

Pi transporter	Putative localization	Tissue expression	Conserved sequence/residue	Reference
AtPHT1;1	Plasma membrane	Roots, hydathodes of cotyledons and leaves, axillary buds, peripheral endosperm of germinating seeds	142 GGDYPLSATIMSE 154	Mudge et al., 2002; Nussaume et al., 2011
AtPHT1;2	Plasma membrane	Roots	142 GGDYPLSATIMSE 154	Mudge et al., 2002; Nussaume et al., 2011
AtPHT1;3	Plasma membrane	Roots	142 GGDYPLSATIMSE 154	Mudge et al., 2002; Nussaume et al., 2011
AtPHT1;4	Plasma membrane	Roots, hydathodes, axillary buds	142 GGDYPLSATIMSE 154	Mudge et al., 2002; Nussaume et al., 2011
AtPht1;5	Plasma membrane	Shoots	142 GGDYPLSATIMSE 154	Mudge et al., 2002; Nussaume et al., 2011
AtPht1;6	Plasma membrane	Flowers, mature pollen grains	143 GGDYPLSATIMSE 155	Mudge et al., 2002; Nussaume et al., 2011
AtPHT1;7	Plasma membrane	Mature pollen grains	142 GGDYPLSATIMSE 154	Mudge et al., 2002; Nussaume et al., 2011
AtPHT1;8	Plasma membrane	Roots	134 GGDYPLSATIMSE 146	Mudge et al., 2002; Nussaume et al., 2011
AtPHT1;9	Plasma membrane	Roots	135 GGDYPLSATIMSE 147	Mudge et al., 2002; Nussaume et al., 2011 Remy et al., 2012
AtPHT2;1	Chloroplast envelope	Shoots	–	Daram et al., 1999; Versaw and Harrison, 2002
AtPHT3;1	Mitochondrial inner membrane	Roots, rosette leaves, and flowers	Cys	Zhu et al., 2012
AtPHT3;2	Mitochondrial inner membrane	Leaves	Cys	Zhu et al., 2012
AtPHT3;3	Mitochondrial inner membrane	Flowers	–	Zhu et al., 2012
AtPHT4;1	Plastid	Shoots	–	Guo et al., 2008 Guo et al., 2008 Guo et al., 2008 Guo et al., 2008 Guo et al., 2008 Guo et al., 2008
AtPHT4;2	Plastid	Roots	–	
AtPHT4;3	Plastid	Veins	–	
AtPHT4;4	Plastid	Shoots	–	
AtPHT4;5	Plastid	Veins	–	
AtPHT4;6	Golgi	Whole plants	–	
AtPHT5;1	Tonoplast	Shoots, roots, pollen, and vascular tissues	–	Liu T.Y. et al., 2016
AtPHT5;2	Tonoplast	Guard cells, vascular tissues and pollen	–	Liu T.Y. et al., 2016
AtPHT5;3	Tonoplast	Shoots, stele, pollen, and vascular tissues	–	Liu T.Y. et al., 2016

### PHT1s

PHT1 proteins are the best studied plant Pi transporters. These proteins have a conserved structure containing 12 transmembrane (TM) domains with a large hydrophilic loop between TM6 and TM7 and both hydrophilic N and C termini locate in the cytoplasm (Raghothama, 1999). All plant PHT1 proteins have the conserved PHT1 signature GGDYPLSATIxSE and in fungi that is GGDYPLSxxIxSE (Karandashov and Bucher, 2005). The first plant *PHT1* gene was cloned from *Arabidopsis* (Muchhal et al., 1996) and exhibited similarities to genes encoding Pi transporters in *Saccharomyces cerevisiae* (*PHO84*; Bun-Ya et al., 1991). Based on protein sequence identity and conserved signature analysis, PHT1 members from several plant species have been identified, including tobacco (Baek et al., 2001), potato (Leggewie et al., 1997; Gordon-Weeks et al., 2003), rice (Paszkowski et al., 2002; Liu et al., 2011; Wang X. et al., 2014; Li et al., 2015), barley (Rae et al., 2003; Preuss et al., 2010, 2011), maize (Liu F. et al., 2016), *Medicago truncatula* (Javot et al., 2007a; Liu et al., 2008), wheat (Liu et al., 2013), soybean (Fan et al., 2013), *Setaria italica* (Ceasar et al., 2014), tomato (Chen et al., 2014), sorghum (Walder et al., 2015), flax (Walder et al., 2015), and poplar (Zhang et al., 2016). The numbers of PHT1 members identified in various plants were listed in **Table 2**. According to the phylogenetic tree, PHT1 proteins were well clustered into three groups. Group I contained most of the PHT1s from all species analyzed and those proteins from dicots were grouped separately with those from monocots. At least one member from all the species analyzed except *Arabidopsis* and *E. salsugineum* was included in group II. Group III contained AtPHT1;8 and AtPHT1;9 from *Arabidopsis* and their orthologs from *E. salsugineum*, OsPT9 and OsPT10 from rice, GmPT3 and GmPT12 from soybean, as well as PtPHT1;13 and PtPHT1;14 from poplar (**Figure 1**). Most of the group I members are reported to be involved in direct Pi uptake from soil such as AtPHT1;1 and AtPHT1;4 (Misson et al., 2004; Shin et al., 2004; Ayadi et al., 2015), OsPT1 (Sun et al., 2012), OsPT2 and OsPT6 (Ai et al., 2009), OsPT4 (Ye et al., 2015; Zhang et al., 2015). It should be pointed out that although AtPHT1;5 and OsPT8 were shown to function in Pi redistribution from source to sink organs (Nagarajan et al., 2011; Li et al., 2015), their roles in Pi uptake cannot be ruled out, considering the tolerance to arsenate (Pi structural analog) of the corresponding mutant plants (Nagarajan et al., 2011; Wang P. et al., 2016). *TaPHT1;2* and *TaPHT1;5* from wheat were strongly induced under Pi-limiting conditions in different cultivars, suggesting their roles in Pi uptake under limited Pi supply. Furthermore, their expression profiles showed genotype-dependent and correlated with plant phosphorus efficiency (Aziz et al., 2014). In poplar, *PtPHT1;2*, *PtPHT1;3*, *PtPHT1;4*, *PtPHT1;6*, *PtPHT1;7*, *PtPHT1;9*, and *PtPHT1;10* clustered in group I were mainly expressed in roots, and *PtPHT1;2*, *PtPHT1;4*, *PtPHT1;6*, and *PtPHT1;8* were greatly induced by Pi starvation (Zhang et al., 2016). Interestingly, *PtPHT1;2* expression was remarkably induced by drought stress, irrespective of the Pi level (Zhang et al., 2016). In addition, seven orthologs of AtPHT1;3 are found in *E. salsugineum*, which was reported more acclimated to Pi limitation than *Arabidopsis* (Velasco et al., 2016). *EsPHT1;4* (Thhalv10016497m) was up-regulated in Pi-deprived *E. salsugineum* plants, suggesting it roles in Pi uptake (Velasco et al., 2016).

**Table 2:** The number of identified PHT1 members in some plants.

Plant	PHT1 members	Reference
*Arabidopsis*	9	Muchhal et al., 1996; Mudge et al., 2002; Shin et al., 2004
Rice	13	Paszkowski et al., 2002; Ming et al., 2005; Liu et al., 2011
Tobacco	5	Baek et al., 2001; Kai et al., 2002; Chen et al., 2007
Potato	5	Leggewie et al., 1997; Rausch et al., 2001; Gordon-Weeks et al., 2003; Nagy et al., 2005
Barley	11	Rae et al., 2003; Mlodzińska and Zboińska, 2016
Maize	13	Liu F. et al., 2016
Tomato	8	Chen et al., 2014
*Medicago truncatula*	6	Javot et al., 2007a; Liu et al., 2008
Soybean	15	Fan et al., 2013
*Setaria italica*	12	Ceasar et al., 2014
Wheat	13	Davies et al., 2002; Sisaphaithong et al., 2012; Shukla et al., 2016
*Eutrema salsugineum*	13	Velasco et al., 2016

Besides absorbing Pi directly from soil, most of vascular plants, including major crops, can obtain Pi from arbuscular mycorrhizal (AM) by exchange of carbohydrates through forming a mycorrhizal association (Javot et al., 2007b). All the PHT1 transporters included in group II (**Figure 1**) except PtPHT1;11 and PtPHT1;12 from poplar, ZmPHT1;10 and ZmPHT1;12 from maize have been reported to be involved in acquisition of Pi from AM (Glassop et al., 2005; Javot et al., 2007a; Tamura et al., 2012; Yang et al., 2012; Duan et al., 2015; Liu F. et al., 2016; Tian et al., 2017). MtPT4 was shown to be essential for Pi acquisition from AM fungus and loss of MtPT4 function even led to premature death of the arbuscules (Javot et al., 2007a). Interestingly, 7 of 13 ZmPHT1 members were clustered in group II (**Figure 1**), implying the importance of AM mediated Pi uptake in maize. ZmPHT1;6 was the best described AM-inducible Pi transporter in maize and its expression was significantly positively correlated with AM colonization rate, concentration of AM biomarkers in roots, Pi uptake and dry weight of shoot (Glassop et al., 2005; Tian et al., 2013). Recently, two studies reported other members including ZmPHT1;2, ZmPHT1;4, ZmPHT1;5, and ZmPHT1;11 were also up-regulated by AM and may participate in Pi uptake through mycorrhizal pathway (Liu F. et al., 2016; Sawers et al., 2017). Moreover, the expression of *ZmPHT1;2*, *ZmPHT1;4*, *ZmPHT1;5*, and *ZmPHT1;6* was correlated with phosphorus acquisition efficiency in maize (Sawers et al., 2017). In fact, in most plant species forming mycorrhizal symbioses at least one mycorrhizal specific or up-regulated *PHT1* gene has been discovered, such as *LePT1* in *Lycopersicon esculentum* (Rosewarne et al., 1999), *StPT3*, *StPT4*, and *StPT5* in potato (Rausch et al., 2001), *AsPT1* in *Astragalus sinicus* (Xie et al., 2013), and *BdPT3*, *BdPT7*, *BdPT12*, and *BdPT13* in *Brachypodium distachyon* (Hong et al., 2012). However, the molecular mechanisms of Pi and carbon transfer in the symbiosis are still largely unknown. In addition, *Arabidopsis* and *E. salsugineum* belong to the minority of plants that cannot associate with AM fungi and our analysis also showed that no *Arabidopsis* PHT1 proteins included in group II, which indicated a certain reliability of predicting unknown gene functions through phylogenetic analysis.

As for group III of PHT1 family, transporters may be involved in Pi uptake as well as Pi allocation from root to shoot, considering the well characterized AtPHT1;8 and AtPHT1;9 (Hamburger, 2002; Lapis-Gaza et al., 2014) and OsPT9 and OsPT10 (Wang X. et al., 2014) in this group. Also, the respective orthologs of AtPHT1;8 and AtPHT1;9 from *E. salsugineum* were included in this group. *PtPHT1;13* and *PtPHT1;14* were predominantly expressed in roots and induced by low Pi conditions, suggesting their roles in Pi uptake (Zhang et al., 2016). *GmPT3* and *GmPT12* were also predominantly expressed in roots. Interestingly, *GmPT3* was up-regulated by N, P, or K deficiency simultaneously in both leaves and roots, suggesting it might be involved in a universal regulation network in response to multiple nutrient deficiencies (Qin et al., 2012).

### PHT2s

PHT2 proteins have a putative topology of 12 TM domains interrupted by a large hydrophilic loop between TM8 and TM9. They have two boxes of eight and nine amino acids located in N- and C-terminal domains, respectively, conserved among eubacteria, archaea, fungi, plants, and animals (Daram et al., 1999). Most of the reported *PHT2;1* genes were predominantly expressed in green tissue and the proteins were located in the chloroplast inner envelope membrane (Versaw and Harrison, 2002; Rausch et al., 2004; Loth-Pereda et al., 2011; Guo et al., 2013; Shi et al., 2013). The PHT2 family has long been thought to have only one member until two members were recently identified in poplar (Zhang et al., 2016). However, though the transcripts of *PtPHT2;1* and *PtPHT2;2* could be detected in various tissues, they were predominantly expressed in roots. Moreover, *PtPHT2;2* significantly up-regulated under low-Pi conditions (Zhang et al., 2016), suggesting its different role from *AtPHT2;1*. Like *AtPHT2;1*, *EsPHT2;1* (Thhalv10003891m) was mainly expressed in leaves and was not regulated by external Pi supply (Velasco et al., 2016). Contrast to *AtPHT2;1 and EsPHT2;1*, *TaPHT2;1* and *OsPHT2;1* (*OsPT14*) were regulated in response to external Pi concentrations (Guo et al., 2013; Shi et al., 2013). Knockdown of TaPHT2;1 significantly reduced Pi concentration in the chloroplast under both sufficient and limited Pi supply, suggesting that TaPHT2;1 is crucial in the mediation of Pi translocation from the cytosol to the chloroplast (Guo et al., 2013). Furthermore, *TaPHT2;1* expression profile was genotype-dependent and correlated with plant phosphorus efficiency (Aziz et al., 2014). Therefore, it was proposed by Aziz et al. (2014) as a marker gene for screening high phosphorus efficiency genotypes. By contrast, overexpression of *OsPHT2;1* (*OsPT14*) in rice increased Pi concentrations in leaves and the plant biomass, suggesting its roles in Pi accumulation in leaves and Pi translocation in plants (Shi et al., 2013).

### PHT3s

PHT3 proteins have two TM α-helices separated by a hydrophilic extramembrane loop, which are conserved in all analyzed mitochondrial transporter proteins, being essential for mitochondrial targeting (Zhu et al., 2012). The first gene encoding mitochondrial Pi transporter (MPT) that belongs to PHT3 family was isolated from birch (*Betula pendula* Roth), named *Mpt1* (Kiiskinen et al., 1997). Afterward, *MPT* cDNAs were continuously isolated and cloned from yeast, soybean, maize, rice, *Arabidopsis*, *Lotus japonicas*, and poplar (Takabatake et al., 1999; Nakamori et al., 2002; Hamel et al., 2004; Sha et al., 2014; Zhang et al., 2016). There are three members in *Arabidopsis*. Interestingly, six transporters from rice and poplar were clustered with AtPHT3 proteins in the phylogenetic tree (**Figure 1**). PHT3 proteins play a critical role in Pi exchange between cytoplasm and mitochondria matrix by Pi/H^+^ symport or Pi/OH^-^ antiport, which is essential for the oxidative phosphorylation of ADP to ATP (Takabatake et al., 1999). Hamel et al. (2004) found that ectopic expression of AtMPT1 (AtPHT3;3) and AtMPT2 (AtPHT3;2) could complement the *S. cerevisiae Δmir1Δpic2* mutant, which was not able to grow on non-fermentable medium at 28 and 36°C, and restored the Pi uptake into mitochondria. The different expression patterns of three *AtMPT* genes imply they play specific roles in various organs or developmental stages (Zhu et al., 2012). For example, overexpression of *AtMPT3 (AtPHT3;1)* caused multiple developmental defects including deformed leaves, dwarfed stature, and reduced fertility through regulating mitochondrial function (Jia et al., 2015). It is worth noting that PHT3s were suggested to be involved in wheat grain development, with high transcript abundance of *TaPHT3;1* in embryo and rachis and *TaPHT3;2* in aleurone (Shukla et al., 2016). Besides, PHT3 transporters were also found to be involved in response to salt and drought stress. *AtMPTs* were up-regulated by high salinity stress in *Arabidopsis* seedlings. And overexpression of *AtMPTs* increased plant sensitivity to salt stress compared with the wild-type plants, which might be through an ATP-dependent pathway and modulation of gibberellin homeostasis (Zhu et al., 2012). Recently, expression of *PtPHT3;2b* and *PtPHT3;3b* from poplar was found to be regulated by drought stress with a Pi level dependent manner (Zhang et al., 2016). To date, knowledge on the molecular mechanisms of PHT3s mediated biological functions in plants is still limited.

### PHT4s

PHT4 family was first characterized and designated by Guo et al. (2008) in *Arabidopsis*. PHT4 proteins share similarity with SLC17/type I Pi transporters. There are six members in PHT4 family in *Arabidopsis* (Guo et al., 2008). The plastid and Golgi apparatus-located PHT4 members in *Arabidopsis* were found to participate in various processes including leaf development, plant defense, and salt response, which were well reviewed by Mlodzińska and Zboińska (2016). Recently, Hassler et al. (2016) found that *pht4;6* mutant *Arabidopsis* plants displayed accelerated senescence induced by darkness. Further investigation indicated that the accelerated dark-induced senescence of *pht4;6* mutant was caused by the reduced *trans*-zeatin concentration, which resulted from cellular Pi starvation (Hassler et al., 2016). Based on genome database searching and phylogenetic analysis, seven putative PHT4 members in rice (Mlodzińska and Zboińska, 2016), eight in poplar (Zhang et al., 2016) and six in wheat (Shukla et al., 2016) were identified recently. Phylogenetic analysis indicated that there are two orthologs of AtPHT4;6 in rice, and two of AtPHT4;1 and AtPHT4;5, respectively, in poplar (**Figure 1**). However, the functions of PHT4s in these plant species are largely unknown yet. *TaPHT4;2* and *TaPHT4;4* in wheat were found to be highly expressed in endosperm during grain development, suggesting their functions in the Pi-allocation within the seed compartments (Shukla et al., 2016). Similarly, all the PHT4 members in rice (*OsPT21*-*26*) were shown to be expressed in all seed tissues (husks, vascular bundle/aleurone, endosperm, and embryo), which suggested that their roles in grain P loading may be not specific (Wang F. et al., 2016). In poplar, *PtPHT4;1a* was up-regulated under low-Pi conditions, while *PtPHT4;1b* and *PtPHT4;5b* were induced under high-Pi (Zhang et al., 2016), suggesting their different roles in response to Pi supply. Interestingly, under high-Pi conditions, *PtPHT4;4* and *PtPHT4;6* were induced by drought stress (Zhang et al., 2016). Most recently, a chromoplast-localized ClPHT4;2 from watermelon was found to be necessary for flesh color development. It was reported that the *ClPHT4;2* expression levels were closely correlated with flesh carotenoid contents among 198 watermelon accessions. Furthermore, down-regulation of *ClPHT4;2* expression in transgenic watermelons decreased the fruit carotenoid accumulation (Zhang et al., 2017). Above all, plant PHT4s play various roles, further investigating their functions, especially in crops, will facilitate the development of crops with desired agronomic traits.

### PHT5s

Plant vacuoles serve as the primary intracellular compartments for Pi storage. Recently, several studies shed light on the underlying molecular mechanisms for vacuolar Pi transport that had long remained unknown. Liu T.Y. et al. (2016) designated *Arabidopsis* SPX-MFS (SYG1/PHO81 /XPR1-Major Facility Superfamily) proteins as PHT5 family, which was also named Vacuolar Phosphate Transporter (VPT), function as vacuolar Pi transporters. In fact, OsSPX-MFS1, OsSPX-MFS2, and OsSPX-MFS3 localized on the tonoplast of rice protoplasts were already functionally characterized before (Wang et al., 2015). Another member OsSPX-MFS4 has not been characterized yet. The roles of PHT5s in Pi transport across the vacuole membrane in *Arabidopsis* and rice were well summarized by Mlodzińska and Zboińska (2016). Further identification and characterization of PHT5s in other crops will be helpful for our understanding on molecular mechanisms of vacuolar Pi transport.

## Regulation of Pi Transporters

Great progress has been made also in deciphering the regulatory mechanism of plant Pi transporters, which has revealed a multi-layered network regulating gene expression, protein activity and protein turnover.

### Transcriptional Regulation

To date, MYB-type, WRKY-type, bHLH-type and other kinds of transcription factors (TF) have been found involved in transcriptional regulation of Pi transporters. PHR1 (phosphate starvation response 1) is a member of the MYB superfamily (Rubio et al., 2001). It was reported that PHR1 regulated expression of *PHT1* at low Pi concentrations through binding to the P1BS (PHR1-binding sequence) or P1BS-like domain, a *cis*-acting element in *PHT1* gene promoters (Rubio et al., 2001; Schünmann et al., 2004a,b). Such interaction was validated in yeast cells where wheat TaPHR1 activated the expression of *TaPHT1;2* through binding to P1BS (Wang et al., 2013). In fact, it has been reported that P1BS is an integrating *cis*-regulatory motif associated with genes that are highly induced by Pi starvation (Bustos et al., 2010). There are usually more than one P1BS in many PHT1 gene promoters, suggesting the important role of PHR1 in regulating *PHT1* expression in response to Pi starvation. For example, P1BS was found in the promoters of 11 of 13 *OsPHT1s*, 8 of 13 *ZmPHT1s*, 10 of 14 *PtPHT1s* and all of the 8 *LePHT1*s in tomato with 1–6 copies (Liu et al., 2011; Chen et al., 2014; Liu F. et al., 2016; Zhang et al., 2016). However, some *PHT1* genes containing P1BS in their promoters were not induced by Pi starvation. For example, OsPT11 and OsPT13 which have 6 and 4 P1BS motif, respectively, are AM-inducible Pi transporters but not involved in Pi starvation response (Yang et al., 2012). Similarly, ZmPHT1;1, LePT4 and LePT5 were not induced by Pi starvation (Chen et al., 2014; Liu F. et al., 2016). Another R2R3 type MYB TF, MYB62, is also induced by Pi starvation (Devaiah et al., 2009). However, the decreased expression of *AtPHT1;1* and *AtPHT1;4* in *MYB62* overexpression lines suggested a negative regulation by MYB62, though the precise mechanism is unclear yet (Devaiah et al., 2009). As a plant-specific TF, WRKY has a conservative WRKYGQK domain at N-terminal and a zinc finger motif. WRKY specifically combines with W-box [TTTGAC(C/T)] in *PHT1* promoters and regulates the expression of *PHT1*. Devaiah et al. (2007) proposed that when inhibiting the expression of WRKY75, a nucleus localized and roots expressed TF, the expression of *AtPHT1;1* and *AtPHT1;4* decreased, suggesting WRKY75 is a positive regulator of these two genes. A similar role was reported for WRKY45, which specifically regulated the expression of At*PHT1;1* (Wang H. et al., 2014; Ding et al., 2016). Recently, Yang et al. (2016) found that up-regulation of TabHLH1, a wheat bHLH-type TF, led to increased expression of *NtPT1* in tobacco and improved plant tolerance to Pi deprivation. Additionally, TaZAT8, a wheat C2H2-ZFP-type TF, was also shown involved in regulation of Pi transporters and mediated low-Pi tolerance through regulating Pi acquisition, ROS homeostasis and root system establishment, however, the regulation mechanism is unclear yet (Ding et al., 2016). Besides, other regulatory elements were also found to regulate the expression of *PHT1* genes, such as SPX3, histone H2A.Z and zinc finger TF ZAT6 (Devaiah et al., 2007; Duan et al., 2008; Smith et al., 2010).

In case of the transcriptional regulation of AM-induced Pi transporters, mycorrhiza transcription factor binding sequence (MYCS) were reported to be involved (Chen A. et al., 2011). In maize, though MYCS was found in the promoter of some AM-inducible *ZmPHT1s*, some *ZmPHT1s* without the MYCS element, such as *ZmPHT1;2*, *ZmPHT1;4*, *ZmPHT1;7*, and *ZmPHT1;9*, also upregulated by AM, implying that new regulation element(s) might exist in the promoter regions of these genes (Liu F. et al., 2016). Most recently, Zhang et al. (2017) identified two TFs (ClbZIP1 and ClbZIP2) from watermelon, binding to the promoter of *ClPHT4;2*, which shed light on our understanding of the regulatory mechanisms of *PHT4s* expression.

### Post-transcriptional and Post-translational Regulation

**FIGURE 2:**
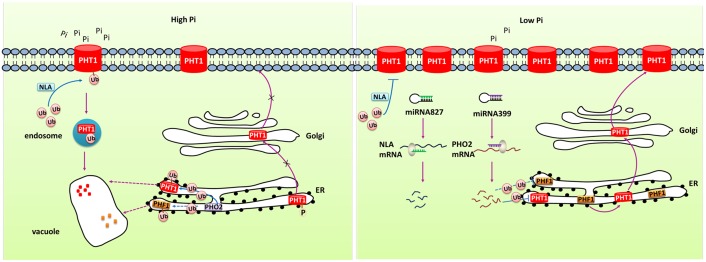
**Simplified models for the post-transcriptional and post-translational regulation of PHT1 transporters.** Under Pi-replete condition, phosphorylation of PHT1 transporters prevents their exit from ER and the subsequent targeting to the PM. NLA and PHO2 direct the ubiquitin-mediated degradation of PHT1 transporters at PM and ER, respectively, to avoid excessive accumulation of Pi. Under Pi deficiency condition, miR827 and miR399 mediate cleavage of *NLA* and *PHO2* transcripts, respectively, thus to increase the amount of PHT1 transporters. PHF1 is accounted for the PHT1 transporters exit from ER and correct targeting to PM.

MicroRNAs (miRNAs) have been reported to regulate gene expression by directing the cleavage of target gene transcript. Many studies have characterized the role of miRNA in regulating Pi homeostasis (**Figure 2**). Under Pi starvation conditions, up-regulated miR399 directed the cleavage of *phosphate2 (PHO2)* mRNA which could degrade PHT1 proteins in *Arabidopsis* (Fujii et al., 2005; Chiou et al., 2006; Kuo and Chiou, 2011). Up-regulation of miR399 in phloem sap was regarded as long-distance signal to regulate Pi homeostasis under Pi deficient conditions. miR399s from some crops including barley, sorghum, tea plant, and cotton were also identified and their predicted targets were PHO2 or UBC24 (Hackenberg et al., 2013; Djami-Tchatchou et al., 2017). miR827, which has been found to target *NLA* (Nitrogen Limitation Adaptation) gene, also regulates Pi homeostasis through proteolysis pathway (Kuo and Chiou, 2011). In addition, the PHT5 members *OsSPX-MFS1* and *OsSPX-MFS2* were also negatively regulated by osa-miR827 (Lin et al., 2010). Furthermore, similarly to the miR399-PHO2 pathway, this pathway is under the control of the central TF OsPHR2 (Wang et al., 2012).

At the post-translational level, some PHT1 proteins can be modified by phosphorylation (**Figure 2**). Ser-514 phosphorylation of AtPHT1;1 C termini prevented its exit from ER suggesting phosphorylation regulated export of AtPHT1;1 from ER and its correct targeting to PM (Bayle et al., 2011). Furthermore, subcellular localization of several PHT1 proteins was revealed to be affected by PHF1 (phosphate transporter traffic facilitator1), a plant unique SCE12 protein (**Figure 2**). It was reported that AtPHT1;1, AtPHT1;2 and AtPHT1;4 transporters could not be targeted to the PM in *phf1* mutant, and phosphate absorptivity of *phf1* mutant was 80% lower than the wild type (González et al., 2005; Bayle et al., 2011). PHF1 was also shown to participate in PHT1 transport from endoplasm to Golgi (González et al., 2005; Bayle et al., 2011). In rice, OsPHF1 had a similar function in efficiently targeting low- and high-affinity Pi transporters to the PM (Chen J. et al., 2011). Hence, PHF1 plays an important role in post-translational regulation of PHT1. Another kind of post-translational regulation is degradation of PHT1 proteins localized at PM under Pi sufficient conditions (**Figure 2**). When Pi-starved seedlings were transferred to sufficient Pi conditions, the number of PHT1 transporters at PM decreased dramatically, indicating a rapid degradation mechanism specifically occurs in Pi-replete conditions (Bayle et al., 2011). An ubiquitin-mediated regulatory pathway has been found to be involved in the degradation of PHT1 members at PM through triggering the endocytosis and vacuolar degradation. The PM-localized NLA protein, a target of miR827, can negatively regulate PHT1 transporters to maintain Pi homeostasis in *Arabidopsis*. Under Pi-sufficient conditions, NLA had a putative ubiquitin ligase activity and mediated the ubiquitination of PHT1 transporters, thus triggered clathrin-dependent endocytosis and PHT1 transporters degradation (Lin et al., 2013). PHO2 is a membrane protein predominately associated with the endoplasmic reticulum (ER) and Golgi. As an ubiquitin-conjugating E2 enzyme, it shared similar mechanism with NLA in degradation of PHT1 proteins at ER including PHT1;1, PHT1;2, and PHT1;3, and likely PHF1 to prevent PHT1s exit from ER and their targeting to PM when the Pi supply is sufficient (Huang et al., 2013). Under Pi deficient condition, miR827 and miR399 mediate post-transcriptional cleavages on the transcripts of NLA and PHO2, thereby relieving the PHT1 transporter degradation and activating Pi uptake as well as root-to-shoot translocation (**Figure 2**). Rice OsNLA also directed the degradation of OsPT members (Lin et al., 2013). Most recently, OsNLA1 was reported to be involved in maintaining phosphate homeostasis in rice by mediating the degradation of OsPT2 and OsPT8. Most importantly, in contrast to the *Arabidopsis* ortholog, the expression of *OsNLA1* was not responding to Pi limitation and also no changes were observed in *OsmiR827* or *OsPHR2*-overexpressing lines. Furthermore, the authors concluded that OsNLA1 does not interact with OsPHO2, suggesting the requirement of additional unknown E2 conjugating enzyme for OsNLA1 mediated degradation of OsPTs (Yue et al., 2017).

Plants response to low phosphorus is a complex signal transduction process, including Pi sense, uptake, transport, translocation and remobilization. Pi transporters and regulatory factors play essential roles in this process. Although a few regulatory factors of PHT1 have been identified as mediators of plant adaptation to low-Pi stress, the accurate regulatory network of PHT1 transporters is still a great challenge to be deciphered. In addition, regulatory mechanisms of PHT2, PHT3, PHT4, and PHT5 family are still unclear as well. Further detailed analysis of these Pi transporter gene promoters is the basis of revelation of the complicated regulatory mechanism. A better understanding of regulation of phosphate transporters will provide novel avenues to improve crop yield with lower Pi inputs

## Potential Applications and Perspectives in Agricultural Production

### To Improve Crop PUE in Pi-Limited Soils

The concerted action of Pi transporters ensures Pi acquisition and distribution among tissues and cytosolic Pi homeostasis. Therefore, engineered alterations of the expression of Pi transporters provide an opportunity to optimize uptake and distribution of Pi in crops to improve yield. Several researches have been illustrated that modulating Pi transporters or regulatory factors could enhance Pi uptake, biomass or crop yields (**Table 3**). For instance, *OsPT4* genetically modified (GM) rice showed increased Pi accumulation in roots and shoots and got significant increase in 1000-grain weight and grain yield per plant compared with wild type under various Pi concentrations (Zhang et al., 2015). Up-regulation of *OsPT6* in soybean or rice also increased the plant biomass, grain weight and grain yield under low-Pi conditions or in field trials (Yan et al., 2014; Zhang et al., 2014). As for TFs, overexpressing *OsWRKY74* in rice resulted in enhanced shoot and root biomass and increased grain yield under low-Pi conditions (Dai et al., 2016). Two regulatory factors (TaPHR1 and TaNFYA-B1) from wheat were also introduced in wheat plant to increase grain yield under low-Pi soils in field trials (Wang et al., 2013; Qu et al., 2015). Recently, knock out *TaPHO2-A1* encoding the negative regulatory factor PHO2, which mediates PHT1 proteins degradation, improved Pi uptake and grain yield under low phosphorus conditions in wheat (Ouyang et al., 2016). These works provided useful candidates for breeding crops with high Pi use efficiency and improved yields. However, many Pi transporters or TFs were shown not to work as well as these proteins in improving plant growth and yields performance, which was usually due to Pi toxicity. For example, overexpression of *OsPT8* in rice resulted in excessive Pi both in roots and shoots and significant growth suppression as well as other Pi toxic symptoms under the high-Pi conditions (Jia et al., 2011) (**Table 3**). Similar results were also observed in *OsPT2*, *OsPT9*, *OsPT10*, *OsPHR2*, and *OsARF12* transgenic plants (Liu et al., 2010; Wang S. et al., 2014; Wang X. et al., 2014) (**Table 3**). Overexpression of miR399 in *Arabidopsis* and tomato also caused Pi toxicity and retarded growth (Fujii et al., 2005; Gao et al., 2009). However, a recent study demonstrated that monitoring expression of *ath-miR399d* by an abiotic stress inducible promoter (*rd29A* promoter) in tomato resulted in plant growth under low temperature and P deficiency conditions (Gao et al., 2015). Therefore, using low Pi or other environment inducible promoters, instead of the constitutive promoters, to control the expression of Pi transporters or their regulatory factors may be a good strategy to avoid Pi toxicity in GM crops.

**Table 3:** Genes encoding Pi transporters and regulatory factors applied in improving Pi accumulation or crop yields.

Genes	Species transformed	Pi accumulation	Growth performance	Pi toxicity symptoms	Field trials	Reference
*OsPT2*	Rice	Excessive Pi in shoot	Leaf toxic symptoms and growth retardation under HP condition	Yes	Yes	Liu et al., 2010
*OsPT4*	Rice	Increased Pi accumulation in roots	Increased 1000-grain weight and grain yield per plant	No	No	Ye et al., 2015; Zhang et al., 2015
*OsPT6*	Rice	Excessive Pi in various tissues, including reproductive tissues	Increased biomass under both HP and LP conditions; increased tillering number, grain weight, and grain yield per plant	No	Yes	Zhang et al., 2014
*OsPT6*	Soybean	High Pi accumulation in leaves, stems and roots	Increased number of pods and seeds, seed weight	No	No	Yan et al., 2014
*OsPT8*	Rice	Excessive Pi in both root and shoot	Significant growth suppression under HP condition	Yes	Yes	Jia et al., 2011
*OsPT9/OsPT10*	Rice	Increased Pi uptake; High Pi concentration under HP and LP condition	Significant reduced biomass of shoots under HP condition	Yes	No	Wang X. et al., 2014
*TaPht1;4*	Wheat	High Pi accumulation in roots and shoots under LP conditions	Increased shoot and root biomass under LP conditions	No	No	Liu et al., 2013
*OsPHT2;1* (*OsPT14*)	Rice	Increased Pi accumulation in leaves under LP condition	Improved biomass under HP and LP conditions	No	Yes	Shi et al., 2013
*OsARF12*	Rice (mutant)	Over accumulated Pi in old leaves	leaf tip necrosis, accompanied by a large number of brown speckles and growth retardation	Yes	No	Wang S. et al., 2014
*OsPHR2*	Rice	Increased Pi accumulation in shoot	With chlorosis or necrosis on the leaf margins, predominantly in mature leaves under HP condition	Yes	Yes	Zhou et al., 2008
*OsWRKY74*	Rice	High P concentration under LP condition	Increased shoot and root biomass, grain weight, tiller number and grain yield under LP conditions	No	No	Dai et al., 2016
*TaNFYA-B1*	Wheat	High P grain concentration under normal, LP and low-nitrogen conditions; increased Pi uptake	Increased grain yield under normal, LP and low-nitrogen conditions	No	Yes	Qu et al., 2015
*TaPHR1*	Wheat	Increased Pi concentration in shoots	Increased grain yield per plant under both HP and LP conditions	No	Yes	Wang et al., 2013
*TaPHO2-A1*	Wheat (knock out)	Increased Pi uptake; High Pi concentration in leaves under HP and LP conditions	Increased grain yield under LP conditions; no adverse effect on grain yield under HP conditions	No	Yes	Ouyang et al., 2016

*HP, High Pi; LP, Low Pi*

All above target genes are involved in Pi uptake from soil, however, it should be pointed out that soil substrate (Pi) availability rather than transporter activity may be the limiting step in such soil conditions. P depletion experiments showed that the P inefficient genotype of rice was able to deplete Pi from nutrient solutions as rapidly and to an equally low level as a P deficiency tolerant genotype (Wissuwa, 2005), which suggested that transporter activity may be not limiting in inefficient genotype. Similarly, overexpressing a high-affinity Pi transporter in barley did not result in improved tolerance to P deficiency (Rae et al., 2004). In fact, several studies comparing gene expression under Pi limitation in different rice genotypes found higher expression of *PHT1s* in the inefficient genotype, which suggested that the Pi deficiency-induced expression of these Pi transporters may be part of the Pi starvation response, but not part of Pi starvation tolerance (Pariasca-Tanaka et al., 2009; Oono et al., 2013).

Taking into account the above problems in using PHT1s to improve crop yields under Pi-limited soils, more attention may be paid to genes encoding intracellular Pi transporters in the future, which play important roles in proper Pi distribution, Pi remobilization, and maintaining cytosolic Pi homeostasis, thus to improve the crop PUE instead of Pi uptake in Pi-limited soils. Consistent with this hypothesis, transgenic rice plants overexpressing *OsPHT2;1* (*OsPT14*) exhibited more Pi accumulation in the top three leaves and panicle axis and significantly higher biomass than the wild type plants under various Pi conditions (Shi et al., 2013) (**Table 3**). In addition, Pi acquisition in crops via AM symbiosis is becoming increasingly important due to limited high grade rock Pi reserves and a demand for environmentally sustainable agriculture. The functional characterization of the interplay between direct and symbiotic Pi uptake, particularly under field conditions, mirroring agricultural practices, would be most valuable and relevant for crops.

### To Improve Crop Growth under Salt and Drought Stress Conditions

Global scarcity of water resources and increasing soil salinity threatens crop productivity worldwide. High soil salinity is usually accompanied by the low availability of many mineral nutrients including P (Grattan and Grieve, 1999). Furthermore, salt and drought stress inhibit the uptake and translocation of mineral nutrients, and P fertilization can increase stress tolerance and dry weight in many plant species (Martinez and Lauchli, 1994; Jin et al., 2006; Waraich et al., 2011; Gao et al., 2015). Therefore, it is of great significance to understand the mechanism of Pi acquisition and utilization under salt and drought stress, thus to improve the crop PUE under abiotic stresses. Efforts can be made in the following three aspects.

Firstly, using identified Pi transporter genes involved in salt or drought response to develop GM crops. So far, several studies have found that Pi transporters are involved in response to salt and drought stress. *Arabidopsis* plants overexpressing *AtPHT3;1*, *AtPHT3;2*, and *AtPHT3;3* were more sensitive to salt stress compared with wild type due to the energy status change and decreased expression of gibberellin (Zhu et al., 2012). In addition, protein *N*-glycosylation has been found associated with plant stress tolerance. AtPHT4;6 was shown to take part in recycling Pi released from glycosylation process and likely to relate to protein *N*-glycosylation. Interestingly, *atpht4;6* mutant was more sensitive to salt stress compared with wild type (Cubero et al., 2009). Hence, intracellular Pi homeostasis is essential for plant salt stress tolerance. Further investigation of the regulatory mechanism of intracellular Pi transporters will be helpful for our understanding on plant Pi acquisition and utilization processes in response to salt stress. Recently, several genes encoding Pi transporters from poplar were found to be regulated by drought stress. *PtPHT1;2* expression was remarkably induced by drought stress, irrespective of the Pi level, whereas changes of *PtPHT1;3*, *PtPHT1;9*, *PtPHT1;11*, *PtPHT1;14*, *PtPHT2;2 PtPHT3;2b*, *PtPHT3;3b*, *PtPHT4;4*, *PtPHT4;6* expression under drought stress showed Pi level dependent. These genes especially those up-regulated by drought stress at low Pi level may contribute to drought tolerance of poplar plants in Pi-limited soils (Zhang et al., 2016).

Secondly, as discussed above, using salt or drought inducible promoters to control the expression of Pi transporters or their regulatory factors may be a potential strategy to improve crop PUE under salt or drought stress conditions.

Last but not least, characterization of Pi transporters in the halophytes will be valuable for our understanding on Pi acquisition and distribution under salinity. Some halophytes have evolved adaptive strategies to high salt and simultaneously low Pi habitat, whereas molecular mechanisms underlying the adaptability remain unclear yet. Comparative studies of the function and expression regulation of halophyte and glycophyte Pi transporters are essential to fully understand the plant Pi acquisition and utilization under salinity. *E. salsugineum* is well known as a model plant for the molecular elucidation of abiotic stress tolerance (Inan and Zhu, 2004; Amtmann et al., 2005; Kant et al., 2006). *E. salsugineum* is also found to be tolerant to nitrogen-limiting conditions by maintaining growth, nitrogen uptake and assimilation (Kant and Rothstein, 2008). Interestingly, it was reported most recently that without significant modification of root architecture, *E. salsugineum* was shown more acclimated to Pi limitation than *Arabidopsis* (Velasco et al., 2016). In this study, *E. salsugineum* and *Arabidopsis* grown either on medium or on soil were subjected to varying Pi treatments. Compared to the enormous changes in root architecture and shoot biomass under low-Pi treatment in *Arabidopsis*, *E. salsugineum* seedlings showed no difference in lateral root density and shoot biomass allocation, while adding NaCl increased lateral root density almost twofold. Furthermore, *E. salsugineum* seedlings had a higher Pi content than *Arabidopsis*. Pi deprived soil-grown *Arabidopsis* plants were stunted with senescing older leaves, whereas *E. salsugineum* plants were visually indistinguishable from Pi-supplemented plants (Velasco et al., 2016). These results suggested a higher Pi uptake and utilization capacity of *E. salsugineum* under low-Pi conditions. Interestingly, seven orthologs of AtPHT1;3 were found in *E. salsugineum* (**Figure 1**), which may be one of the mechanisms underlying the higher Pi uptake in *E. salsugineum* than *Arabidopsis*. Further investigations indicated that some Pi starvation associated genes including *EsPHT1;4* were up-regulated in Pi-deprived *E. salsugineum* plants, while *EsPHR1*, *EsWRKY75*, and *EsRNS1* showed constitutively higher expression levels relative to those in *Arabidopsis* regardless of external Pi (Velasco et al., 2016). These results suggested that specific regulatory mechanism of Pi transporters might be one of the adaptive strategies of *E. salsugineum* to high salt and simultaneously low Pi habitat. Further comparative expression patterns analysis of Pi transporters between *E. salsugineum* and *Arabidopsis* under salt stress and low-Pi conditions will help to identify members that are involved in Pi uptake and distribution under salinity conditions, which can be valuable for improving crop yields challenged by increasing soil salinization and shrinking farmland.

## Conclusion

Plant Pi transporters play an essential role in Pi acquisition and distribution. PHT1 transporters are mainly involved in Pi uptake from soil and translocation, and they can be regulated at transcriptional, post-transcriptional and post-translational levels. PHT2/3/4/5 family participate in Pi transport within subcellular compartments, playing important roles in maintaining cytosolic Pi homeostasis, however, their regulatory mechanism is largely unknown yet. Although some PHT1 transporters and regulatory factors have been confirmed useful candidates for breeding crops with improved yields in Pi-limited soils, induced Pi toxicity as a result of PT overexpression is still a serious problem limiting the application of these proteins. Furthermore, many studies suggested that soil substrate (Pi) availability rather than transporter activity may be the limiting step in Pi-limited soil. In these respects, more attention should be paid to intracellular Pi transporters in the future to improve the crop PUE instead of Pi uptake in Pi-limited soils. In addition, three potential strategies for improving crop PUE under salt or drought stress conditions are provided, among which *E. salsugineum* is suggested as an appropriate system for studying plant Pi uptake and utilization under salinity and for identifying candidate genes that may be valuable for breeding crops with high PUE under salinized soils. Besides, Pi transporter may also be potential candidates for application in phytoremediation of arsenic contaminated soils, considering the roles of many PHT1s in plant AsV uptake.

## Author Contributions

YL and SL conceived the manuscript. DW and SL wrote the manuscript. PJ and YL revised and checked the manuscript. All authors approved the final manuscript.

## Conflict of Interest Statement

The authors declare that the research was conducted in the absence of any commercial or financial relationships that could be construed as a potential conflict of interest.
