# Supplementary material for: Roles, Regulation, and Agricultural Application of Plant Phosphate Transporters
Source: Front Plant Sci. 2017 May 18;8:817. doi: 10.3389/fpls.2017.00817 (PMC5435767; doi:10.3389/fpls.2017.00817)
Supplement: Supplementary file 1 [file Table_1.DOCX]

Supplementary Material

**Roles, Regulation and Agricultural Application of Plant Phosphate Transporters**

*Duoliya Wang****^†^****^12^, Sulian Lv****^†^****^1^, Ping Jiang^1^,* *Yinxin Li*^1^*

*^1^Key Laboratory of Plant Molecular Physiology, Institute of Botany, Chinese Academy of Sciences, Beijing 100093, China*

^2^*University of Chinese Academy of Sciences*

**^†^**The two authors contribute equally to this work.

* Correspondence

Yinxin Li

[yxli@ibcas.ac.cn](mailto:yxli@ibcas.ac.cn)

# Supplementary Tables

**Table S1 Accession numbers or locus IDs of all used proteins for phylogenetic tree construction in Fig.1.**

| Transporter name | Protein length (AA) | Accession number | Locus |
| --- | --- | --- | --- |
| AtPHT1;1 | 524 | Q8VYM2 | At5g43350 |
| AtPHT1;2 | 524 | Q96243 | At5g43370 |
| AtPHT1;3 | 521 | O48639 | At5g43360 |
| AtPHT1;4 | 534 | Q96303 | At2g38940 |
| AtPHT1;5 | 542 | Q8GYF4 | At2g32830 |
| AtPHT1;6 | 516 | Q9ZWT3 | At5g43340 |
| AtPHT1;7 | 526 | Q494P0 | At3g54700 |
| AtPHT1;8 | 541 | Q9SYQ1 | At1g20860 |
| AtPHT1;9 | 532 | Q9S735 | At1g76430 |
| AtPHT2;1 | 587 | Q38954 | At3g26570 |
| AtPHT3;1 | 375 | Q9FMU6 | At5g14040 |
| AtPHT3;2 | 363 | Q7DNC3 | At3g48850 |
| AtPHT3;3 | 309 | Q9M2Z8 | At2g17270 |
| AtPHT4;1 | 529 | O82390 | At2g29650 |
| AtPHT4;2 | 535 | Q7XJR2 | At2g38060 |
| AtPHT4;3 | 519 | Q66GI9 | At3g46980 |
| AtPHT4;4 | 541 | Q8GX78 | At4g00370 |
| AtPHT4;5 | 471 | Q3E9A0 | At5g20380 |
| AtPHT4;6 | 428 | Q9FKV1 | At5g44370 |
| AtPHT5;1 | 708 | Q2V4F9 | At1g63010 |
| AtPHT5;2 | 707 | Q9T050 | At4g11810 |
| AtPHT5;3 | 700 | Q93ZQ5 | At4g22990 |
| OsPT1 | 527 | Q8H6H4 | Os03g05620 |
| OsPT2 | 528 | Q8GSD9 | Os03g05640 |
| OsPT3 | 526 | Q7XDZ7 | Os10g30770 |
| OsPT4 | 538 | Q8H6H2 | Os04g10750 |
| OsPT5 | 548 | Q7X7V2 | Os04g10690 |
| OsPT6 | 534 | Q8H6H0 | Os08g45000 |
| OsPT7 | 526 | Q8H6G9 | Os03g04360 |
| OsPT8 | 541 | Q8H6G8 | Os10g30790 |
| OsPT9 | 582 | Q8H6G7 | Os06g21920 |
| OsPT10 | 552 | Q69T94 | Os06g21950 |
| OsPT11 | 555 | Q94DB8 | Os01g46860 |
| OsPT12 | 541 | Q8H074 | Os03g05610 |
| OsPT13 | 508 | Q7XRH8 | Os04g10800 |
| OsPT14 | 572 | Q6ZH86 | Os02g38020 |
| OsPT15 | 368 | O80414 | Os02g52860 |
| OsPT16 | 374 | Q10NP8 | Os03g15690 |
| OsPT17 | 360 | Q7XV24 | Os04g37600 |
| OsPT18 | 368 | Q69TX3 | Os06g10810 |
| OsPT19 | 349 | Q0J198 | Os09g28160 |
| OsPT20 | 323 | AK241216 | Os09g38100 |
| OsPT21 | 529 | Q9SDI4 | Os01g17240 |
| OsPT22 | 519 | Q8W0H5 | Os01g63290 |
| OsPT23 | 535 | Q53WP9 | Os05g37820 |
| OsPT24 | 591 | Q652N5 | Os09g39680 |
| OsPT25 | 428 | Q53P54 | Os11g08370 |
| OsPT26 | 439 | Q2QWW7 | Os12g07970 |
| OsSPX-MFS1 | 696 | Q0JAW2 | Os04g48390 |
| OsSPX-MFS2 | 697 | Q6EPQ3 | Os02g45520 |
| OsSPX-MFS3 | 698 | Q658H5 | Os06g03860 |
| OsSPX-MFS4 | 570 |  | Os09g34990 |
| PtPHT1;1 | 523 |  | Potri.002G038900 |
| PtPHT1;2 | 539 |  | Potri.005G223600 |
| PtPHT1;3 | 539 |  | Potri.005G223500 |
| PtPHT1;4 | 535 |  | Potri.001G318500 |
| PtPHT1;5 | 536 |  | Potri.010G071700 |
| PtPHT1;6 | 536 |  | Potri.010G071600 |
| PtPHT1;7 | 536 |  | Potri.010G072000 |
| PtPHT1;8 | 436 |  | Potri.010G071500 |
| PtPHT1;9 | 534 |  | Potri.005G175500 |
| PtPHT1;10 | 533 |  | Potri.005G175700 |
| PtPHT1;11 | 528 |  | Potri.015G022800 |
| PtPHT1;12 | 531 |  | Potri.019G061900 |
| PtPHT1;13 | 528 |  | Potri.002G005500 |
| PtPHT1;14 | 528 |  | Potri.005G256100 |
| PtPHT2;1 | 320 |  | Potri.008G186600 |
| PtPHT2;2 | 590 |  | Potri.010G046300 |
| PtPHT3;1a | 366 |  | Potri.001G322300 |
| PtPHT3;1b | 374 |  | Potri.017G060800 |
| PtPHT3;2a | 364 |  | Potri.012G105100 |
| PtPHT3;2b | 359 |  | Potri.015G104400 |
| PtPHT3;3a | 314 |  | Potri.004G207200 |
| PtPHT3;3b | 318 |  | Potri.005G098800 |
| PtPHT4;1a | 518 |  | Potri.001G249800 |
| PtPHT4;1b | 520 |  | Potri.009G043800 |
| PtPHT4;2 | 513 |  | Potri.016G111000 |
| PtPHT4;3 | 528 |  | Potri.001G248200 |
| PtPHT4;4 | 580 |  | Potri.014G085700 |
| PtPHT4;5a | 543 |  | Potri.006G062300 |
| PtPHT4;5b | 553 |  | Potri.018G121600 |
| PtPHT4;6 | 442 |  | Potri.009G168200 |
| GmPT1 | 533 | FJ814697 | Glyma02g00840 |
| GmPT2 | 539 | FJ814696 | Glyma03g31950 |
| GmPT3 | 516 | FJ814701 | Glyma07g34870 |
| GmPT4 | 533 | JQ518269 | Glyma10g00950 |
| GmPT5 | 521 | FJ814694 | Glyma10g04230 |
| GmPT6 | 502 | FJ814693 | Glyma10g33020 |
| GmPT7 | 536 | FJ814695 | Glyma10g33030 |
| GmPT8 | 519 | FJ814700 | Glyma13g08720 |
| GmPT9 | 525 | FJ814698 | Glyma14g28780 |
| GmPT10 | 529 | FJ814699 | Glyma14g36650 |
| GmPT11 | 539 | JQ518270 | Glyma19g34710 |
| GmPT12 | 506 | FJ814692 | Glyma20g02660 |
| GmPT13 | 536 | FJ789662 | Glyma20g34610 |
| GmPT14 | 527 | JQ518271 | Glyma20g34620 |
| GmMPT | 375 | O80412 | Glyma19g101100 |
| ZmPHT1;1 | 539 |  | GRMZM2G326707 |
| ZmPHT1;2 | 537 |  | GRMZM2G139639 |
| ZmPHT1;3 | 543 |  | GRMZM2G112377 |
| ZmPHT1;4 | 516 |  | GRMZM2G170208 |
| ZmPHT1;5 | 509 |  | GRMZM2G041595 |
| ZmPHT1;6 | 554 |  | GRMZM5G881088 |
| ZmPHT1;7 | 587 |  | GRMZM2G075870 |
| ZmPHT1;8 | 535 |  | GRMZM2G045473 |
| ZmPHT1;9 | 541 |  | GRMZM2G154090 |
| ZmPHT1;10 | 597 |  | GRMZM2G159075 |
| ZmPHT1;11 | 517 |  | GRMZM2G009779 |
| ZmPHT1;12 | 590 |  | GRMZM2G009800 |
| ZmPHT1;13 | 524 |  | GRMZM2G070087 |
| ZmMPT | 366 | O80413 |  |
| MtPT1 | 537 | O22301 |  |
| MtPT2 | 533 | O22302 |  |
| MtPT3 | 533 | EF016358 |  |
| MtPT4 | 528 | Q8GSG4 |  |
| MtPT5 | 538 | EF016359 |  |
| MtPT6 | 541 | B2G3Q0 |  |
| MtPHT2;1 | 574 | Q8GTR3 |  |
| TaPHT1;1 | 533 |  | AJ830009 |
| TaPHT1;2 | 525 |  | AY293828 |
| TaPHT1;5 | 535 | W5FKS5 |  |
| TaPHT1;11 | 554 |  | AB753270 |
| TaPHT2;1 | 568 |  | AY293827 |
| TaPHT3;3 | 369 | W5BGN6 |  |
| TaPHT4;1 | 522 | A0A077RRV5 |  |
| TaPHT4;4 | 610 | W5DN82 |  |
| TaPHT4;5 | 428 | A0A077S0J7 |  |
| EsPHT1;3 | 521 | V4LQE0 | Thhalv10003183m.g |
| Thhalv10003187m | 521 | V4NF38 | Thhalv10003187m.g |
| Thhalv10003186m | 521 |  | Thhalv10003186m.g |
| EsPHT1;4 | 534 | V4P021 | Thhalv10016497m.g |
| EsPHT1;5 | 543 | V4M6F3 | Thhalv10016483m.g |
| Thhalv10003190m | 516 | V4L318 | Thhalv10003190m.g |
| Thhalv10010274m | 537 | V4LMN8 | Thhalv10010274m.g |
| EsPHT1;8 | 525 | V4L4M4 | Thhalv10009424m.g |
| Thhalv10018375m | 537 | V4K909 | Thhalv10018375m.g |
| Thhalv10003336m | 490 | V4LXT9 | Thhalv10003336m.g |
| Thhalv10003182m | 521 |  | Thhalv10003182m.g |
| Thhalv10003340m | 521 |  | Thhalv10003340m.g |
| Thhalv10003184m | 521 |  | Thhalv10003184m.g |
| EsPHT2;1 | 586 | V4MMR5 | Thhalv10003891m.g |
